# Impact of Reporter Type on Signal Detection of Cancer Therapy-Induced Alopecia: A Hypothesis-Generating Study Using the FDA Adverse Event Reporting System

**DOI:** 10.3390/ph19030445

**Published:** 2026-03-10

**Authors:** Airi Yajima, Yoshihiro Uesawa

**Affiliations:** Department of Medical Molecular Informatics, Meiji Pharmaceutical University, 2-522-1 Noshio, Kiyose 204-8588, Tokyo, Japan

**Keywords:** FAERS, disproportionality analysis, alopecia, reporter bias, healthcare professional stratification, reporting odds ratio, hypothesis-generating study

## Abstract

**Background/Objectives**: Cancer therapy-induced alopecia (CTIA) profoundly affects patients’ quality of life. This study conducted a disproportionality analysis of CTIA using the FDA Adverse Event Reporting System (FAERS) database to provide an overview of drug-specific signal distributions by systematically evaluating the impact of reporter type on CTIA signal detection. **Methods**: FAERS data from January 2004 to September 2024 were analyzed to extract alopecia-related Preferred Terms included under the Medical Dictionary for Regulatory Activities High Level Term “Alopecias.” Reporting odds ratios (RORs) were calculated to assess disproportionality. A primary analysis including all reports and a stratified analysis restricted to reports submitted by healthcare professionals (HCPs) were performed. No individual case-level clinical review was conducted. **Results**: Approximately 90% of alopecia reports were associated with female patients, and approximately 40% of these reports were linked to breast cancer. In the disproportionality analysis including all reporters, the highest ROR [95% confidence interval (CI)] was observed for docetaxel [58.31 (57.46–59.17)]. In the analysis restricted to HCP reports, the highest ROR was observed for vismodegib [23.92 (21.86–26.17)], whereas that for docetaxel markedly decreased to 3.68 (3.48–3.89). For molecular targeted agents, statistically significant signals were maintained even in the HCP-restricted analysis. **Conclusions**: Reporter characteristics substantially influence the detection of alopecia signals, with patients amplifying signals reflecting psychological harm and HCPs amplifying signals reflecting pharmacological plausibility. These findings should be interpreted as hypothesis-generating and warrant further validation using prospective or clinical datasets.

## 1. Introduction

Changes in physical appearance associated with cancer treatment, particularly alopecia, profoundly affect patients’ self-perception, interpersonal relationships, and health-related quality of life (HRQoL) [1,2,3]. Hair both symbolizes health status and reflects social roles and cultural identity, making hair loss a major source of psychosocial stress [1,2,3].

Alopecia associated with cancer therapy is classically categorized into acute, extensive hair loss caused by cytotoxic chemotherapy [chemotherapy-induced alopecia (CIA)] [4], and chronic diffuse alopecia resulting from alterations in the hormonal milieu [endocrine therapy-induced alopecia (EIA)] [5]. In recent years, characteristic alopecia patterns associated with molecular targeted therapies and immunotherapies have been reported [6], highlighting drug-specific manifestations of hair loss.

CIA arises from cytotoxic injury to rapidly proliferating hair matrix keratinocytes, resulting in anagen effluvium. Taxanes, such as docetaxel and paclitaxel, are strongly associated with severe alopecia and, in some instances, permanent CIA (pCIA) [7,8,9]. Greater cumulative exposure to docetaxel has been linked to an increased risk of persistent alopecia [7], and genetic susceptibility—including polymorphisms in the *ABCB1* gene—may further influence susceptibility to pCIA [10].

Endocrine therapies may cause chronic diffuse alopecia by altering hormonal regulation of the hair cycle and disrupting the estrogen–androgen balance [11]. Targeted agents, including Hedgehog pathway inhibitors and cyclin-dependent kinase 4/6 (CDK4/6) inhibitors, interfere with hair follicle signaling pathways and cell cycle control, resulting in distinct, drug-specific patterns of alopecia [12,13,14,15,16,17]. These mechanistic variations help explain the clinical heterogeneity of alopecia and provide biological plausibility for assessing such associations in pharmacovigilance databases.

Advances in cancer treatment have led to prolonged survival; thus, patients can experience long-term treatment-related adverse effects even after achieving remission. Although alopecia does not directly affect survival, it has been suggested to reduce patients’ willingness to continue treatment and impair adherence [3], and it is increasingly recognized as a clinically relevant issue that might influence long-term treatment outcomes. However, alopecia deemed mild or predictable is often insufficiently captured in clinical trials, making it difficult to comprehensively assess its occurrence and characteristics in real-world clinical practice.

Spontaneous reporting systems (SRSs) represent valuable resources for comprehensively collecting safety information for drugs, and they provide complementary findings to clinical trials by capturing rare adverse events or symptoms that are difficult to detect in controlled settings. Nevertheless, SRS data are inherently susceptible to reporting bias, as reporting depends on the characteristics and motivations of reporters. Consequently, mild or expected adverse events tend to be underreported [18,19,20]. Moreover, adverse events related to HRQoL are frequently reported by patients but less frequently reported by healthcare professionals (HCPs) [21,22,23].

Against this background, the present study evaluated signals of cancer therapy-induced alopecia associated with antineoplastic and endocrine therapies using an exploratory, hypothesis-generating disproportionality analysis based on the FDA Adverse Event Reporting System (FAERS). In addition, we quantitatively assessed the impact of reporter type (HCPs vs. non-HCPs) on reporting odds ratios (RORs). Signal patterns across drug classes were further visualized using volcano plots, and integrated interpretations were attempted from both pharmacological and reporting bias perspectives. Given the exploratory and hypothesis-generating nature of this study, analyses were conducted under the hypothesis that restricting reports to those submitted by HCPs would result in lower RORs for appearance-related, HRQoL-associated adverse events than analyses including all reports.

## 2. Results

### 2.1. Baseline Characteristics of the Analytical Dataset

Baseline characteristics for the all-reporter dataset are presented in Table 1a.

In total, 76,580 cases were included in the analysis. Sex information was available for 62,565 cases. Of these, 56,378 cases (90.11%) involved female patients, whereas 6097 cases (9.75%) involved male patients. Sex was classified as unknown in 90 cases (0.14%). Among the 45,821 cases for which age information was available, the median age was 57 years [interquartile range (IQR) = 47–66]. Body weight information was available for 22,371 cases, and the median weight was 75 kg (IQR = 64–91 kg).

Country information was available for 76,308 cases. The largest number of reports originated from the U.S. (52,928 cases, 69.36%), followed by Canada (11,800 cases, 15.46%), the UK (1683 cases, 2.21%), Germany (1331 cases, 1.74%), and Japan (1113 cases, 1.46%). Meanwhile, country information was missing for 1335 cases (1.75%) As the numbers of reports from Germany was comparable to that of reports with missing country information, Japan was included to present the top six reporting countries.

Indication information was available for 71,767 cases. In the all-reporter dataset, breast cancer-related Preferred Terms (PTs) predominated, including “Breast cancer female” (13,615 cases, 18.97%), “Breast cancer” (7635 cases, 10.64%), “Breast cancer metastatic” (3025 cases, 4.22%), and “Triple negative breast cancer” (699 cases, 0.97%). Collectively, breast cancer-related indications accounted for more than 35% of all cases. “Rheumatoid arthritis” was the second-most frequent indication, reported in 9270 cases (12.92%).

### 2.2. Alopecia-Related PTs Included in the Analysis

In total, 22 PTs were classified under the High-Level Term (HLT) “Alopecias.” After excluding PTs as specified in the exclusion criteria, 13 PTs representing alopecia types for which drug involvement could not be excluded were included in the analysis. The complete list of included PTs is provided in Supplementary Table S1. Seborrhoeic alopecia was not present in the analytical dataset. The most frequent reported term was alopecia, followed by madarosis and alopecia areata (Table 2a).

### 2.3. Drug-Specific Reporting Frequencies

The top 25 drugs with the highest number of alopecia-related reports are presented in Table 3a. The number of reports was highest for docetaxel, followed by methotrexate and cyclophosphamide.

### 2.4. Distribution of Reporter Characteristics

**Figure 1:**
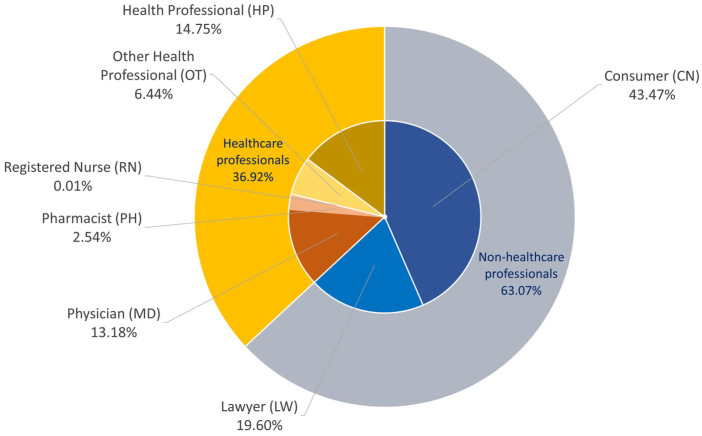
Case-based distribution of reporter types for alopecia-related adverse event reports in FAERS.

**Figure 2:**
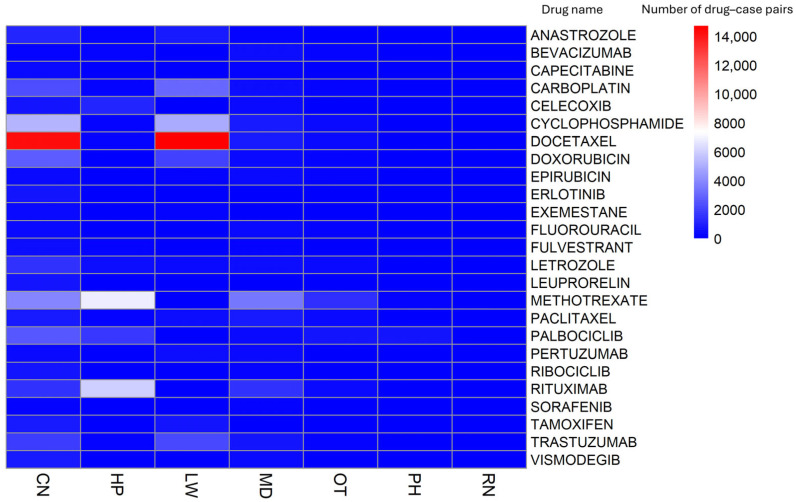
Case-based heatmap of alopecia-related adverse event reports by drug and reporter occupation.

Reporter occupation was aggregated on a case basis (primary ID level) for cases reporting alopecia-related events. Reports submitted by consumers and legal professionals accounted for more than half of all cases, whereas reports from HCPs accounted for 36.92% of cases (Figure 1). When drug- and occupation-specific reporting counts were visualized using a heatmap, certain drugs exhibited marked clustering in reports from consumers and legal professionals (Figure 2). For docetaxel, reports from consumers and legal professionals were particularly prominent, with 14,148 reports submitted by consumers and 14,720 reports submitted by legal professionals (Supplementary Table S2).

To further probe temporal changes in reporter mix, we examined quarterly trends in reporter composition around the initiation of U.S. mass litigation related to docetaxel. As shown in Supplementary Figures S1 and S2, reports from non-HCPs increased sharply after 2016 Q4, whereas reports from HCPs remained comparatively stable. This shift was evident both in absolute report counts and in proportional composition.

Percentages were calculated on the basis of unique primary case IDs. The outer ring represents reporter categories (HCPs vs. non-HCPs), whereas the inner ring presents the distribution of FAERS occupation codes, including physicians, pharmacists, registered nurses, health professionals, other health professionals, consumers, and lawyers. HCPs comprised physicians, pharmacists, registered nurses, health professionals, and other HCPs.

This heatmap visualizes the distribution of alopecia-related adverse event cases across the top 25 drugs with the highest reporting frequency stratified by reporter occupation. Each cell represents the number of unique primary cases (primary IDs) in which a given drug–alopecia combination was reported by a specific reporter occupation category.

Reporter occupation was defined on the basis of the occupation code recorded in the FAERS DEMO table and classified as consumers (CN), lawyers (LW), physicians (MD), pharmacists (PH), registered nurses (RN), other health professionals (OT), and health professionals (HP). Color intensity indicates the number of cases, with darker colors representing a higher number of reported cases.

HCPs include MDs, PHs, RNs, OTs, and HPs not otherwise specified, while non-HCPs include CNs and LWs.

### 2.5. Disproportionality Analysis in the All-Reporter Dataset

Disproportionality analysis using RORs was conducted to evaluate the association between each target drug and alopecia-related adverse events. All included alopecia-related PTs were treated as a single outcome group for ROR calculation. Several antineoplastic agents exhibited strong associations with alopecia-related events, most notably docetaxel (ROR = 58.31, 95% CI = 57.46–59.17, *p* < 0.001). Elevated RORs were also observed for vismodegib (ROR: 19.35, 95% CI: 18.24–20.52, *p* < 0.001) and trastuzumab (ROR = 8.23, 95% CI = 8.00–8.47, *p* < 0.001). Conversely, some drugs, such as leuprorelin (ROR = 0.99, 95% CI = 0.93–1.06, *p* = 0.764), did not exhibit statistically significant associations with alopecia (Table 4a).

### 2.6. Results of the Stratified Analysis Restricted to HCPs

#### 2.6.1. Patient Characteristics in the HCP-Restricted Dataset

The baseline characteristics of cases reported by HCPs are summarized in Table 1b. Female patients predominated, and the median age was 58 years (IQR = 45–67). Indication information was available for 28,190 cases. The most frequent indication was rheumatoid arthritis (7740 cases, 27.46%), followed by product used for unknown indication (5687 cases, 20.17%), breast cancer (1728 cases, 6.13%), breast cancer metastatic (1251 cases, 4.44%), and breast cancer female (1160 cases, 4.12%).

#### 2.6.2. Alopecia-Related PTs Reported by HCPs

Table 2b presents the distribution of alopecia-related PTs reported by HCPs. Alopecia was the most frequently reported PT, accounting for 49,986 reports, followed by madarosis (754 reports) and alopecia areata (469 reports).

#### 2.6.3. Drug-Specific Reporting Frequencies in HCP Reports

In the dataset restricted to reports submitted by HCPs, drug-specific reporting frequencies for alopecia-related adverse events are summarized in Table 3b.

Methotrexate was the most frequently reported drug (11,863 reports), followed by rituximab (7929 reports) and palbociclib (3470 reports). Endocrine therapies and molecular targeted agents—including letrozole, fulvestrant, ribociclib, and vismodegib—were also among the most frequently reported drugs in the HCP-restricted dataset.

In contrast, docetaxel, which ranked highest in the all-reporter analysis, occupied a lower position in the HCP-restricted dataset (1408 reports). Other cytotoxic chemotherapeutic agents, such as cyclophosphamide, carboplatin, and doxorubicin, were reported but were not among the top-ranked drugs.

Overall, the pattern of frequently reported medications differed between the HCP-restricted and all-reporter datasets, suggesting variations in reporting patterns according to reporter type.

#### 2.6.4. Disproportionality Analysis in the HCP-Restricted Dataset

Disproportionality analysis based on alopecia-related PTs was conducted using reports submitted by HCPs only. The results for drugs with high reporting frequencies are presented in Table 4b. Several drugs demonstrated statistically significant associations with alopecia-related events, including vismodegib (ROR = 23.92, 95% CI = 21.86–26.17, *p* < 0.001) and palbociclib (ROR = 11.34, 95% CI = 10.94–11.75, *p* < 0.001). By contrast, a significant association was noted for cisplatin (ROR = 1.05, 95% CI = 0.96–1.15, *p* = 0.326). Oxaliplatin featured an ROR below unity (ROR = 0.87, 95% CI = 0.79–0.95, *p* = 0.003), suggesting no positive association with alopecia.

#### 2.6.5. Differences in RORs According to Reporter Type

**Figure 3:**
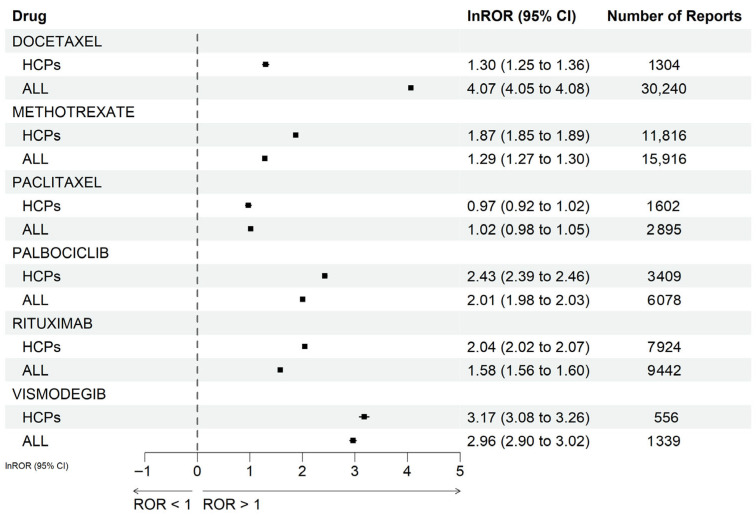
Comparison of drug-specific lnRORs for alopecia-related adverse events between all reporters and HCPs.

Figure 3 compares changes in RORs for major drugs between the all-reporter dataset and the HCP-restricted dataset. For docetaxel, lnROR values were markedly higher in the all-reporter analysis than in the HCP-restricted analysis, suggesting potential overestimation of the association because of reporting bias.

The forest plots present the lnROR and 95% CIs for major drugs associated with alopecia-related adverse events. Results derived from reports submitted by HCPs and those submitted by all reporters (ALL) are presented for comparison.

#### 2.6.6. Volcano Plot

**Figure 4:**
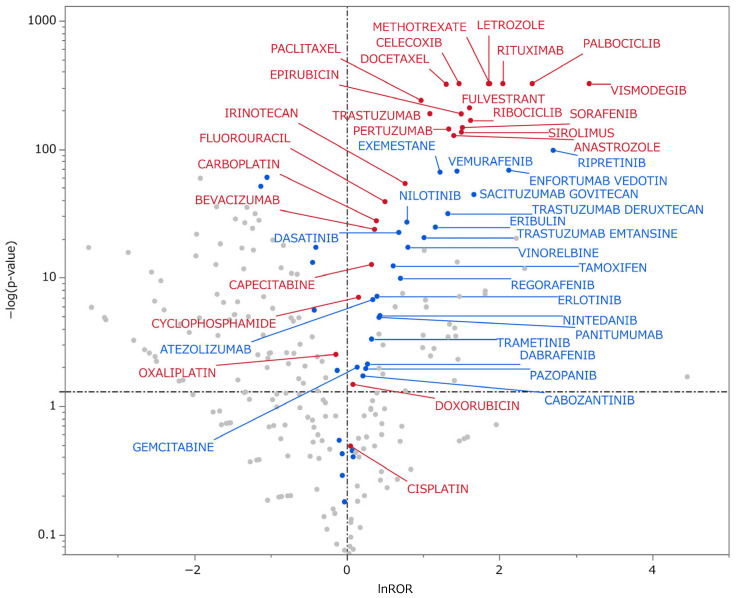
Volcano plot of L01 and L02 drugs for alopecia-related adverse events based on reports from HCPs.

Volcano plots depicting the lnROR and −log_10_(*p*) were generated to present the results by simultaneously visualizing effect size and statistical significance. These plots allow intuitive identification of drugs that demonstrate both positive disproportionality (lnROR > 0) and statistical significance (*p* < 0.05) [24]. As presented in Figure 4, drugs demonstrating statistically suggestive associations with alopecia included 23 of the top 25 drugs by reporting frequency that satisfied the criteria of lnROR > 0 and *p* < 0.05 (Table 4b), as well as 22 additional drugs with at least 100 reports that met the same statistical thresholds. In total, 45 drugs were identified as potential alopecia-related signals.

To minimize reporter-related bias and enhance pharmacological plausibility, the volcano plot analysis was conducted using HCP-only reports.

Each point represents an individual drug. The *x*-axis represents the lnROR, reflecting the strength of association with alopecia-related adverse events. The *y*-axis represents −log_10_(*p*) obtained from Fisher’s exact test, indicating statistical significance. Red-colored and labeled plots correspond to drugs ranked among the top 25 by reporting frequency (Table 4b). Blue points indicate drugs with 100 or more reports. Among these, labeled blue plots located in the first quadrant (lnROR > 0 and −log10(*p*) > 1.3) indicate drugs with statistically significant disproportionality (ROR > 1, *p* < 0.05) and are interpreted as potential alopecia-related safety signals. The vertical dashed line corresponds to lnROR = 0 (ROR = 1), while the horizontal dashed line represents *p* = 0.05 (−log10(*p*) = 1.3). 

## 3. Discussion

The most prominent and clinically meaningful finding of this study was the identification of a substantial reporter-related bias in alopecia-related safety signals derived from the FAERS database, particularly for docetaxel. Although docetaxel exhibited an extremely high ROR in the overall dataset, this signal markedly attenuated when the analysis was restricted to reports submitted by HCPs. Such attenuation may indicate that the magnitude of the spontaneous reporting signal exceeds clinically observed patterns, particularly for pCIA, which has been reported after taxane chemotherapy—especially docetaxel—in observational cohorts and retrospective surveys [7,8].

A retrospective study has demonstrated higher rates of persistent alopecia with docetaxel than with paclitaxel [8]. Beyond differences in incidence, pCIA represents a clinically meaningful and psychologically distressing condition with considerable implications for quality of life [3,25]. This discrepancy appears to be largely attributable to the disproportionate number of reports submitted by consumers and legal professionals, potentially reflecting ongoing litigation in the U.S. concerning permanent chemotherapy-induced alopecia. These findings underscore that the reporter type can substantially distort signal estimates, particularly for adverse events that are visually apparent and emotionally distressing, such as alopecia, and therefore are more likely to stimulate patient-driven or legally motivated reporting.

### 3.1. Characteristics of Alopecia Reporting

In this study, we analyzed reporting patterns of cancer treatment-related alopecia using the FAERS database and examined the influence of reporter characteristics on signal detection. Alopecia-related adverse events were defined using 13 PTs classified under the Medical Dictionary for Regulatory Activities (MedDRA) HLT “Alopecias” after excluding congenital, infectious, radiation-induced, and localized forms of alopecia. Target drugs were defined as those classified under Anatomical Therapeutic Chemical (ATC) categories L01 and L02.

The inclusion of both antineoplastic and endocrine therapies was based on the biological rationale that cytotoxic injury and hormonal alterations can affect the hair cycle and hair follicle stem cell function, thereby contributing to alopecia [5,26,27,28,29,30]. This approach was particularly relevant for breast cancer populations, in which chemotherapy and endocrine therapy are frequently combined or administered sequentially [28,29,31].

Previous FAERS-based analyses also identified strong alopecia signals for docetaxel [32], consistent with the findings from our all-reporter dataset.

The marked discrepancy in RORs between paclitaxel and docetaxel in our overall dataset might reflect differences in pCIA risk.

In the HCP-restricted analysis, a substantial proportion of reports involved rheumatologic indications. Methotrexate, a cornerstone disease-modifying antirheumatic drug, is widely used in long-term treatment regimens [33,34] and is well known to cause alopecia through folate antagonism [35,36]. Methotrexate-associated alopecia is generally reversible, and it can be mitigated by folic acid supplementation or dose adjustment [37]. Chronic disease management and structured follow-up by rheumatologists might facilitate more systematic adverse event reporting by HCPs. Conversely, CIA often anticipated and transient, potentially leading to underreporting by clinicians. This reporting asymmetry might explain why consumer reports are enriched for oncology-related alopecia, whereas HCP reports more frequently involve rheumatologic drugs, reflecting structural differences in reporting culture across disease areas.

### 3.2. Reporter Type and Bias in Reporting Patterns

This study demonstrated that consumer and legal professional reports constituted the majority of alopecia-related FAERS submissions, whereas reports from HCPs accounted for approximately one-third of all alopecia-related submissions. This pattern suggests that appearance-related and quality of life (QoL) adverse events, although highly salient to patients, might be deprioritized by clinicians who focus primarily on life-threatening toxicities.

Reports concerning docetaxel were principally submitted by consumers and legal professionals (Figure 2). In the U.S., multidistrict litigation (MDL 2740) concerning permanent alopecia allegedly caused by docetaxel is ongoing [38,39,40,41,42,43]. These legal actions highlight a disconnect between patient expectations and clinical communication regarding the potential permanence of alopecia.

Within this context, docetaxel featured an exceptionally high ROR in the all-reporter analysis but a substantially weaker signal decreased in the HCP-restricted analysis. This pattern strongly suggests that reporter bias inflated the signal [18,44]. In randomized phase III clinical trials, docetaxel and paclitaxel have demonstrated generally comparable overall safety profiles. However, docetaxel has been associated with a higher incidence of grade 3/4 non-hematologic toxicities [45]. In our HCP-restricted analysis, the RORs for these agents were more comparable, aligning more consistent with the overall safety findings reported in randomized trials.

Although HCP reports tend to offer higher diagnostic accuracy, patient reports provide valuable insights into symptom burden, daily functioning, and emotional impact. Prior studies have indicated that patient reports more frequently capture QoL-related adverse events and psychological distress [46,47]. Therefore, stratifying analyses by reporter type might allow both clinically robust signal detection and a more comprehensive understanding of patient experience.

The temporal patterns observed in Supplementary Figures S1 and S2 are consistent with the possibility of stimulated reporting following the initiation of mass litigation. The disproportionate increase in reports from non-HCPs suggests that external socio-legal events may have influenced reporting behavior.

### 3.3. HCP-Stratified Analysis and Visualization Using Volcano Plots

This approach highlighted persistent and statistically significant alopecia signals for targeted therapies and endocrine agents, including Hedgehog pathway inhibitors (vismodegib), CDK4/6 inhibitors (palbociclib, ribociclib), and endocrine therapies (letrozole, anastrozole, exemestane, fulvestrant, tamoxifen). These findings are consistent with established mechanisms involving hair follicle cycling and hormonal regulation [12,15,16,17,48].

Signals detected for monoclonal antibodies (trastuzumab, pertuzumab, and rituximab) likely reflect combination regimens and disease context rather than direct drug effects. HER2-targeted antibodies are commonly administered with taxanes or anthracyclines, which are primary drivers of CIA [49,50,51]. Rituximab is frequently used in both lymphoma and autoimmune diseases, often in combination with cytotoxic agents or MTX [52,53,54]. Therefore, the elevated RORs observed for rituximab in the HCP-restricted analysis likely reflect the attribution complexities inherent to FAERS rather than a direct causal effect. Because ROR indicates reporting disproportionality rather than causality [18], these signals must be interpreted cautiously. Drug-specific, indication-stratified, and monotherapy-focused analyses are required for more definitive risk assessment.

### 3.4. Sex Differences in Alopecia: Clinical, Psychosocial, and QoL Implications

In addition to reporter-type differences, a prominent finding of this study was the marked predominance of female reports. Approximately 90% of alopecia reports in this study involved female patients, with breast cancer accounting for nearly 40% of cases. Sex-based differences in adverse drug reaction (ADR) reporting are well recognized. Prior studies have consistently shown that women tend to report ADRs more frequently than men across various drug classes [55,56,57]. In the present study, however, the magnitude of female predominance for alopecia substantially exceeds that typically reported for general ADR patterns, suggesting that alopecia may be especially sensitive to sex-related influences (Table 1a,b).

One possible explanation is the predominance of breast cancer indications among female patients. However, our stratified analyses suggest that indication alone does not fully account for the observed disparity (Table 1b). This finding suggests that sex-related differences in the perception, salience, and reporting of hair loss may contribute to the observed pattern. Hair is closely linked to gender identity and social roles, and alopecia may be perceived as particularly identity-threatening among female patients. Conversely, under-recognition or underreporting among male patients cannot be excluded and warrants further investigation, especially given societal norms that may normalize hair loss in men.

Biological factors may also play a role. Women exhibit sex-related differences in pharmacokinetics, including variations in hepatic enzyme activity, body fat distribution, and drug clearance, all of which can influence systemic drug exposure and susceptibility to ADRs [58,59,60]. Although direct evidence linking these physiological differences to CIA severity is limited, differential exposure to cytotoxic agents could plausibly contribute to sex-related variability in both adverse event experience and reporting.

Beyond reporting patterns, alopecia carries significant clinical and psychosocial implications. Persistent alopecia following chemotherapy has been reported in a subset of patients [61,62,63], leading to long-term impairment of self-esteem, social functioning, and HRQoL [3,25].

Alopecia has been identified as a major barrier to treatment initiation and adherence [3,64], and qualitative studies suggest that some patients perceive treatment-related hair loss as equally, or even more, distressing than surgical body image changes, such as those resulting from mastectomy [65]. Patient-reported outcome measures, such as the EORTC QLQ-BR42, the updated breast cancer-specific QoL module that replaced the provisional BR45 version, assess distress related to hair loss [66,67], underscoring its clinical relevance.

Importantly, alopecia is not merely a cosmetic concern but a multidimensional experience affecting identity, femininity, privacy, and social visibility. Preventive strategies, such as scalp cooling, have displayed efficacy in reducing CIA [68,69], whereas therapeutic options, including topical and oral minoxidil, remain investigational [70,71,72]. For EIA and pCIA, evidence-based interventions are limited, potentially exacerbating the associated psychosocial burden.

Psychosocial interventions, particularly appearance care programs, have emerged as important supportive strategies. Programs such as Look Good Feel Better aim to preserve self-image through cosmetic counseling and have demonstrated benefits in emotional well-being and self-efficacy [73,74,75]. From both a clinical and pharmacovigilance perspective, these findings underscore the importance of sex-sensitive counseling and safety monitoring. First, safety assessment and signal interpretation should account for potential sex-related differences in adverse event occurrence and reporting behavior. Second, clinicians should provide sex-sensitive counseling, ensuring that male patients are explicitly informed about the possibility and impact of treatment-related hair loss. Directly addressing male patients may help reduce underreporting and unmet supportive care needs. Finally, future clinical trials should incorporate sex-stratified safety analyses and patient-reported outcomes to more accurately capture differential adverse event experiences between sexes.

Overall, integrating biological, psychosocial, and reporting perspectives is essential for comprehensive management of alopecia in oncology.

### 3.5. Study Limitations and Future Directions

This study carried several limitations inherent to SRSs. FAERS is subject to underreporting, missing data, and reporting bias, and it does not allow causal inference. Detailed information on dose, treatment duration, and combination regimens is often unavailable, limiting granular risk assessment. Non-serious adverse events, such as alopecia, might be disproportionately influenced by patient perception and external stimuli, and the absence of statistical disproportionality does not equate to the absence of risk.

Additionally, FAERS primarily reflects U.S. reporting patterns, which can limit generalizability. Future research integrating electronic health records and prospective cohort studies is needed to better characterize temporal relationships, risk factors, and intervention effects. Prospective evaluations of preventive and supportive strategies, particularly for persistent alopecia, are warranted to inform patient-centered care and personalized risk mitigation.

## 4. Materials and Methods

### 4.1. Data Source and Study Period

This study utilized FAERS, a spontaneous reporting database maintained by the U.S. FDA. Reports submitted between January 2004 and September 2024 (October 2024 data release) were included in the analysis [76]. Data curation, including coding, mapping, and data cleaning, was conducted by ArkMS Inc. (Tokyo, Japan; https://www.arkms.co.jp/; accessed on 30 November 2025). This process included normalization of drug names registered in FAERS (standardization to generic names and consolidation of synonyms and spelling variants) and assignment of ATC codes to each drug.

### 4.2. Terminology for Target Drugs and Adverse Events

Drug classes were defined using the ATC Classification System developed by the WHO Collaborating Centre for Drug Statistics Methodology [77]. In this study, drugs classified under the second-level ATC categories L01 (antineoplastic agents) and L02 (endocrine therapy) were included.

The Medical Dictionary for Regulatory Activities (MedDRA) is a standardized international medical terminology with a five-level hierarchical structure: System Organ Class, High Level Group Term, HLT, PT, and Lowest-Level Term.

To comprehensively and clinically appropriately capture alopecia-related adverse events, this study targeted all PTs classified under the HLT “Alopecias” based on MedDRA version 27.1. A complete list of the PTs included in the analysis is provided in Supplementary Table S1.

Drugs recorded as “primary suspect” or “secondary suspect” in the FAERS DRUG table were included, whereas those classified as “concomitant” or “interaction” were excluded. Additionally, PTs related to radiation therapy or localized injection-site reactions, or alopecia with clearly non-drug-related etiologies, such as congenital or infectious causes, were excluded.

### 4.3. Data Extraction and Integration

FAERS DRUG, adverse reaction information (REAC), patient demographic data (DEMO), and indication information (INDI) tables were used. These tables were integrated using the primary ID as a common key, and an inner join with the INDI table was additionally performed using the drug sequence number.

**Figure 5:**
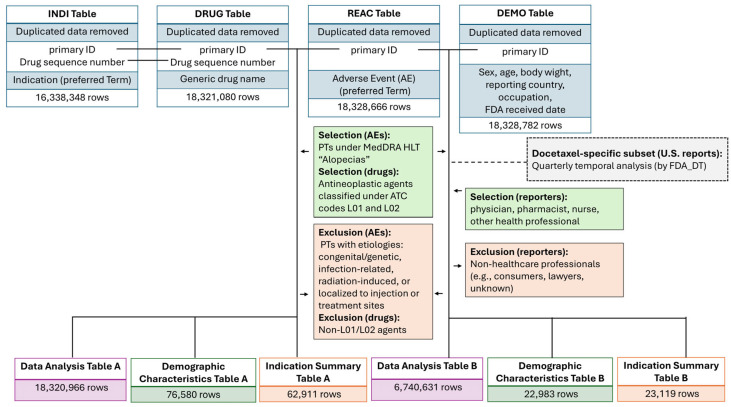
Flowchart of data curation and construction of analysis datasets.

To construct the analytical datasets, the DRUG and REAC tables were merged by primary ID to identify cases (primary IDs) that included combinations of target drugs (ATC categories L01 and L02) and alopecia-related adverse events (MedDRA HLT “Alopecias”). Next, the DEMO table was linked to the extracted primary IDs to append patient background information, including sex, age, body weight, reporting country, and reporter type (occupation code). Subsequently, the INDI table was merged using the primary ID and corresponding drug sequence number to aggregate the indication data. Through this series of integration steps, analytical dataset encompassing drug–adverse event–patient background–INDI was constructed (Figure 5).

Based on this integrated dataset, case-based (primary ID–level) aggregations were performed according to the analytical objectives, and tables were created to visualize reporting distributions by drug and reporter occupation. To ensure analytical consistency, age and body weight were standardized. Age recorded in days, weeks, months, or decades was converted to years. For decade-based entries, the midpoint value (e.g., 35 years for “30 s”) was assigned as a representative value. Body weight recorded in pounds was converted to kilograms. These standardized variables were used to generate descriptive statistics of patient characteristics and analytical tables for subsequent disproportionality and reporter-stratified analyses.

The requirements for ethical approval and informed consent were waived by the Ethics Committee of Meiji Pharmaceutical University because this study used anonymized data from a publicly available database and did not involve identifiable human subjects.

This flowchart illustrates the data curation process used to construct the analysis datasets from FAERS. DRUG, REAC, DEMO, and INDI tables were integrated after removing duplicated records. Alopecia-related adverse events were identified using PTs under the MedDRA HLT “Alopecias,” and antineoplastic agents classified under ATC codes L01 and L02 were selected. Reports submitted by healthcare professionals (physicians, pharmacists, nurses, and other HCPs) and non-HCPs were distinguished. Based on this process, six datasets were generated: analysis dataset A, patient background table A, and indication table A, which included all reporters, and analysis dataset B, patient background table B, and indication table B, which were restricted to HCP reports.

In addition, a docetaxel-specific subset of U.S. reports was constructed for supplementary quarterly temporal analyses based on the FDA received date (FDA_DT).

### 4.4. Assessment of Reporting Frequency and Reporter Characteristics

To identify drugs associated with alopecia, the reporting frequencies of drugs and alopecia-related adverse events included in the analytical dataset were summarized, and drugs with high reporting frequencies were extracted. To examine reporter characteristics and differences in reporting patterns according to reporter type, heatmaps were generated to visualize the distribution of reports by reporter occupation for the top 25 drugs with the highest reporting frequencies.

To aggregate reporter characteristics, alopecia-related PTs were integrated and treated as a single adverse event group (alopecia-related events). Reporter occupation (occupation code) was evaluated on a case basis (primary ID level). The reporter occupation associated with each primary ID was classified according to the occupation code recorded in the DEMO table. Both the analysis of reporter composition and the generation of heatmaps were performed using case-based aggregation.

To further explore potential temporal changes in reporting patterns, quarterly trends in reporter composition and total report counts were summarized descriptively for U.S. FAERS reports of docetaxel-associated alopecia. Both the proportional and absolute number of reports were calculated for each calendar quarter to assess changes that might be related to external events. These analyses were conducted on a case-based (primary ID–level) dataset and are presented in Supplementary Figures S1 and S2.

### 4.5. Disproportionality Analysis

The primary outcome was the differences in signal detection according to reporter type. Secondary analyses included a comprehensive assessment of alopecia-related signals, visualized using volcano plots.

For each target drug, all alopecia-related events were combined into a single outcome group, and RORs with 95% CIs were calculated to evaluate the strength of association (Table 5).

Disproportionality analyses were conducted on a report-level basis using a case/non-case approach. For each drug–event combination, a 2 × 2 contingency table was constructed on the basis of unique FAERS reports identified by primary IDs. To avoid duplicate counting because of follow-up reports, records were deduplicated, ensuring that each primary ID was counted only once. All analyses were therefore performed on a one report per primary ID basis. In this study, the term “case” refers to a unique FAERS report after deduplication rather than a clinical case defined by CASEID.

Each cell represents the number of cases (primary IDs) classified as follows:

(a) cases in which the drug of interest (antineoplastic agents classified under ATC codes L01 or L02) was reported as a suspect drug and alopecia (MedDRA HLT “Alopecias”) was reported;
(b) cases in which the drug of interest was reported as a suspect drug but alopecia was not reported;
(c) cases in which drugs other than the drug of interest were reported as suspect drugs and alopecia was reported;
(d) cases in which drugs other than the drug of interest were reported as suspect drugs and alopecia was not reported.

Based on this analysis, RORs and their associated 95% CIs were calculated, and statistical significance was assessed using Fisher’s exact test. In this study, all ROR calculations were performed using a case-based approach.

### 4.6. Stratified Analysis Restricted to HCPs

Given the potential impact of reporter characteristics on ADR reporting patterns and signal detection, a stratified analysis restricted to reports submitted by HCPs, including physicians, pharmacists, nurses, and other healthcare workers, was performed.

HCP reports were identified using the occp_cod variable in the DEMO table of the FAERS database. In this study, reports with occupation codes MD, PH, RN, HP, and OT were classified as submissions from HCPs.

### 4.7. Statistical Analysis

In both the all-reporter and HCP-restricted analyses, RORs and 95% CIs were calculated, and statistical significance was assessed using Fisher’s exact test. When zero cells were present, the Haldane–Anscombe correction (adding 0.5 to each cell) was applied to stabilize estimates [78].

In addition, volcano plots were generated to visualize the results of the HCP-restricted analysis. Natural logarithms of the RORs (lnROR) and −log_10_(*p*-values) were calculated, and signals were defined as lnROR > 0 and −log_10_(*p*) > 1.3 (i.e., ROR > 1 and *p* < 0.05).

Data processing and statistical analyses were performed using JMP Pro 18.2 (SAS Institute Inc., Cary, NC, USA) and Python (version 3.12.3; pandas version 2.3.1, SciPy version 1.13.1; https://www.python.org/). Forest plots and heatmaps were generated using R (version 4.5.1; The R Foundation for Statistical Computing, Vienna, Austria) and the RStudio environment (PBC, Boston, MA, USA). The “forestploter” package was used for forest plots, and the “pheatmap” package was used for heatmaps. Given the exploratory nature of this study, the significance threshold was set at *p* < 0.05.

## 5. Conclusions

This study conducted a comprehensive disproportionality analysis using a large-scale spontaneous reporting database to evaluate alopecia signals associated with antineoplastic and endocrine therapies. By incorporating a stratified analysis restricted to reports submitted by HCPs, we demonstrated that certain drugs exhibit substantial differences in the reporting proportion of alopecia between all reporters and HCPs.

These findings provide database-level evidence that alopecia, although typically regarded as a medically non-severe adverse event, represents a clinically meaningful issue for patients, exerting a considerable psychological burden and potentially influencing motivation to continue treatment. This underscores the importance of recognizing cancer treatment-related alopecia as both a QoL-related adverse event and a clinical challenge that can affect treatment adherence and psychosocial well-being.

Furthermore, visualization using volcano plots enabled the comprehensive and intuitive identification of drugs associated with higher alopecia risk. This approach might provide a practical foundation for considering preventive strategies and psychosocial support prior to treatment initiation. Collectively, these findings contribute to the development of a patient-centered drug safety evaluation framework and provide scientific support for establishing comprehensive care systems addressing alopecia in cancer therapy.

## Figures and Tables

**Table 1:** Baseline characteristics of cases included in the analysis dataset.

	a. All Reporter Types			b. Healthcare Professionals Only			
	(n = 76,580)			(n = 27,838)			
Characteristics		No.	(%)		No.	(%)	
Gender							
	Data available	62,565		Data available	24,560		
	Female	56,378	90.11%	Female	21,488	87.49%	
	Male	6097	9.75%	Male	3015	12.28%	
	Unknown	90	0.14%	Unknown	57	0.23%	
Age (years old)							
	Data available	45,821		Data available	17,802		
	less than 30	1190	2.60%	less than 30	710	3.99%	
	30–39	2474	5.40%	30–39	818	4.60%	
	40–49	10,267	22.41%	40–49	4457	25.04%	
	50–59	12,355	26.96%	50–59	3954	22.21%	
	60–69	11,624	25.37%	60–69	4190	23.54%	
	70–79	6190	13.51%	70–79	2861	16.07%	
	80 or more	1721	3.76%	80 or more	812	4.56%	
	Median (IQR)	57(47–66)		Median (IQR)	58(45–67)		
Body weight (kg)							
	Data available	22,371		Data available	6434		
	less than 40	169	0.76%	less than 40	112	1.74%	
	40–49	802	3.59%	40–49	337	5.24%	
	50–59	2855	12.76%	50–59	890	13.83%	
	60–69	4829	21.59%	60–69	1620	25.18%	
	70–79	4294	19.19%	70–79	1024	15.92%	
	80–89	3181	14.22%	80–89	635	9.87%	
	90–99	3321	14.85%	90–99	1445	22.46%	
	100 or more	2920	13.05%	100 or more	371	5.77%	
	Median (IQR)	75(64–91)		Median (IQR)	72(62–93)		
Reported countries (Top6)							
	Data available	76,308		Data available	27,711		
	United States	52,928	69.36%	United States	10,429	37.64%	
	Canada	11,800	15.46%	Canada	9741	35.15%	
	United Kingdom	1683	2.21%	Germany	1079	3.89%	
	Country Not Specified	1335	1.75%	Japan	1004	3.62%	
	Germany	1331	1.74%	United Kingdom	944	3.41%	
	Japan	1113	1.46%	Italy	800	2.89%	
Indication (pt_term, Top15)							
	Data available	71,767			28,190		
	Product used for an unknown indication	14,038	19.56%	Rheumatoid Arthritis	7740	27.46%	
	Breast Cancer Female	13,615	18.97%	Product used for an unknown indication	5687	20.17%	
	Rheumatoid Arthritis	9270	12.92%	Breast Cancer	1728	6.13%	
	Breast Cancer	7635	10.64%	Breast Cancer Metastatic	1251	4.44%	
	Breast Cancer Metastatic	3025	4.22%	Breast Cancer Female	1160	4.12%	
	Chemotherapy	1355	1.89%	Chronic Myeloid Leukemia	456	1.62%	
	Invasive ductal breast carcinoma	1020	1.42%	Basal Cell Carcinoma	407	1.44%	
	Chronic Myeloid Leukemia	985	1.37%	Non-Small-Cell Lung Cancer	357	1.27%	
	Basal Cell Carcinoma	976	1.36%	Ovarian Cancer	277	0.98%	
	Lung Neoplasm Malignant	742	1.03%	Psoriatic Arthropathy	222	0.79%	
	Ovarian Cancer	734	1.02%	Renal Cell Carcinoma	197	0.70%	
	Triple-negative breast cancer	699	0.97%	Colorectal Cancer Metastatic	194	0.69%	
	Non-Small-Cell Lung Cancer	651	0.91%	Arthritis	193	0.69%	
	Prostate Cancer	587	0.82%	Neoplasm Malignant	189	0.67%	
	Gastrointestinal Stromal Tumor	529	0.74%	Lung Neoplasm Malignant	184	0.65%	

Reports submitted by HCPs included physicians, pharmacists, nurses, health professionals, and other HCPs, as defined by occupation codes in the DEMO table.

**Table 2:** Number of reports by PTs for alopecia-related adverse events.

(a) All Reporter Types		(b) Healthcare Professionals Only	
Adverse Event	Number of Reports	Adverse Event	Number of Reports
Alopecia	135,323	Alopecia	49,986
Madarosis	17,871	Madarosis	754
Alopecia areata	1203	Alopecia areata	469
Alopecia totalis	294	Diffuse alopecia	176
Diffuse alopecia	278	Alopecia totalis	130
Hypotrichosis	168	Alopecia scarring	74
Alopecia scarring	105	Androgenetic alopecia	52
Androgenetic alopecia	79	Hypotrichosis	40
Alopecia universalis	61	Alopecia universalis	32
Lichen planopilaris	18	Lichen planopilaris	14
Follicular mucinosis	15	Follicular mucinosis	13
Non-scarring alopecia	4	Non-scarring alopecia	4

Reports submitted by HCPs included physicians, pharmacists, nurses, health professionals, and other HCPs, as defined by occupation codes in the DEMO table.

**Table 3:** Top 25 drugs with the highest number of alopecia-related reports.

	(a) All Reporter Types		(b) Healthcare Professionals Only	
	(n = 155,419)		(n = 51,744)	
Rank	Drug Name	Number of Reports	Drug Name	Number of Reports
1	Docetaxel	37,305	Methotrexate	11,863
2	Methotrexate	16,038	Rituximab	7929
3	Cyclophosphamide	14,557	Palbociclib	3470
4	Rituximab	9454	Celecoxib	1728
5	Carboplatin	8258	Paclitaxel	1623
6	Doxorubicin	6698	Letrozole	1507
7	Trastuzumab	6539	Docetaxel	1408
8	Palbociclib	6182	Cyclophosphamide	1374
9	Letrozole	3850	Trastuzumab	1091
10	Paclitaxel	3233	Carboplatin	979
11	Anastrozole	3050	Bevacizumab	909
12	Celecoxib	2681	Fluorouracil	838
13	Tamoxifen	2104	Doxorubicin	779
14	Pertuzumab	1960	Epirubicin	614
15	Fluorouracil	1487	Fulvestrant	614
16	Vismodegib	1382	Capecitabine	588
17	Fulvestrant	1290	Pertuzumab	585
18	Epirubicin	1272	Vismodegib	571
19	Bevacizumab	1210	Irinotecan	539
20	Ribociclib	1192	Ribociclib	481
21	Capecitabine	994	Anastrozole	469
22	Exemestane	961	Sorafenib	469
23	Leuprorelin	926	Cisplatin	440
24	Erlotinib	923	Sirolimus	434
25	Sorafenib	826	Oxaliplatin	410

Reports submitted by HCPs included physicians, pharmacists, nurses, health professionals, and other HCPs, as defined by occupation codes in the DEMO table.

**Table 4:** Associations of drugs with alopecia-related adverse events.

**(a** **) All Reporter-Type**					
**Drugs**	**ROR**	**95%CI [Lower, Upper]**	**lnROR**	**95%CI [Lower, Upper]**	***p*-Value**
DOCETAXEL	58.31	[57.46, 59.17]	4.07	[4.05, 4.08]	*p* < 0.001
VISMODEGIB	19.35	[18.24, 20.52]	2.96	[2.90, 3.02]	*p* < 0.001
TRASTUZUMAB	8.23	[8.00, 8.47]	2.11	[2.08, 2.14]	*p* < 0.001
ANASTROZOLE	8.02	[7.70, 8.35]	2.08	[2.04, 2.12]	*p* < 0.001
TAMOXIFEN	7.60	[7.23, 7.99]	2.03	[1.98, 2.08]	*p* < 0.001
PALBOCICLIB	7.44	[7.25, 7.64]	2.01	[1.98, 2.03]	*p* < 0.001
LETROZOLE	6.60	[6.38, 6.84]	1.89	[1.85, 1.92]	*p* < 0.001
CYCLOPHOSPHAMIDE	6.59	[6.46, 6.72]	1.89	[1.87, 1.90]	*p* < 0.001
PERTUZUMAB	6.19	[5.88, 6.51]	1.82	[1.77, 1.87]	*p* < 0.001
CARBOPLATIN	5.94	[5.79, 6.09]	1.78	[1.76, 1.81]	*p* < 0.001
EPIRUBICIN	5.13	[4.83, 5.44]	1.63	[1.58, 1.69]	*p* < 0.001
DOXORUBICIN	4.97	[4.83, 5.10]	1.60	[1.58, 1.63]	*p* < 0.001
RIBOCICLIB	4.91	[4.63, 5.21]	1.59	[1.53, 1.65]	*p* < 0.001
RITUXIMAB	4.86	[4.75, 4.96]	1.58	[1.56, 1.60]	*p* < 0.001
FULVESTRANT	4.57	[4.32, 4.84]	1.52	[1.46, 1.58]	*p* < 0.001
EXEMESTANE	4.22	[3.94, 4.52]	1.44	[1.37, 1.51]	*p* < 0.001
SORAFENIB	3.95	[3.68, 4.24]	1.37	[1.30, 1.44]	*p* < 0.001
METHOTREXATE	3.62	[3.56, 3.68]	1.29	[1.27, 1.30]	*p* < 0.001
PACLITAXEL	2.76	[2.66, 2.87]	1.02	[0.98, 1.05]	*p* < 0.001
CELECOXIB	2.10	[2.02, 2.18]	0.74	[0.70, 0.78]	*p* < 0.001
ERLOTINIB	2.00	[1.87, 2.14]	0.69	[0.63, 0.76]	*p* < 0.001
FLUOROURACIL	1.59	[1.51, 1.68]	0.46	[0.41, 0.52]	*p* < 0.001
BEVACIZUMAB	1.06	[1.00, 1.12]	0.06	[0.00, 0.12]	*p* = 0.042
CAPECITABINE	1.05	[0.99, 1.12]	0.05	[−0.01, 0.12]	*p* = 0.114
LEUPRORELIN	0.99	[0.93, 1.06]	−0.01	[−0.08, 0.06]	*p* = 0.764
**(b** **) Healthcare Professionals Only**					
**drug**	**ROR**	**95%CI [lower, upper]**	**lnROR**	**95%CI [lower, upper]**	***p*-value**
VISMODEGIB	23.92	[21.86, 26.17]	3.17	[3.08, 3.26]	*p* < 0.001
PALBOCICLIB	11.34	[10.94, 11.75]	2.43	[2.39, 2.46]	*p* < 0.001
RITUXIMAB	7.72	[7.53, 7.91]	2.04	[2.02, 2.07]	*p* < 0.001
METHOTREXATE	6.50	[6.37, 6.63]	1.87	[1.85, 1.89]	*p* < 0.001
LETROZOLE	6.39	[6.06, 6.74]	1.86	[1.80, 1.91]	*p* < 0.001
RIBOCICLIB	5.06	[4.62, 5.55]	1.62	[1.53, 1.71]	*p* < 0.001
FULVESTRANT	4.99	[4.60, 5.41]	1.61	[1.53, 1.69]	*p* < 0.001
SORAFENIB	4.54	[4.14, 4.99]	1.51	[1.42, 1.61]	*p* < 0.001
SIROLIMUS	4.49	[4.08, 4.94]	1.50	[1.41, 1.60]	*p* < 0.001
EPIRUBICIN	4.47	[4.12, 4.85]	1.50	[1.42, 1.58]	*p* < 0.001
CELECOXIB	4.35	[4.14, 4.56]	1.47	[1.42, 1.52]	*p* < 0.001
ANASTROZOLE	4.06	[3.70, 4.46]	1.40	[1.31, 1.49]	*p* < 0.001
PERTUZUMAB	3.80	[3.49, 4.13]	1.33	[1.25, 1.42]	*p* < 0.001
DOCETAXEL	3.68	[3.48, 3.89]	1.30	[1.25, 1.36]	*p* < 0.001
TRASTUZUMAB	2.97	[2.79, 3.16]	1.09	[1.03, 1.15]	*p* < 0.001
PACLITAXEL	2.64	[2.51, 2.78]	0.97	[0.92, 1.02]	*p* < 0.001
IRINOTECAN	2.14	[1.97, 2.34]	0.76	[0.68, 0.85]	*p* < 0.001
FLUOROURACIL	1.65	[1.54, 1.76]	0.50	[0.43, 0.57]	*p* < 0.001
CARBOPLATIN	1.47	[1.38, 1.57]	0.39	[0.32, 0.45]	*p* < 0.001
BEVACIZUMAB	1.44	[1.35, 1.54]	0.36	[0.30, 0.43]	*p* < 0.001
CAPECITABINE	1.38	[1.27, 1.50]	0.32	[0.24, 0.41]	*p* < 0.001
CYCLOPHOSPHAMIDE	1.17	[1.10, 1.23]	0.15	[0.10, 0.21]	*p* < 0.001
DOXORUBICIN	1.08	[1.01, 1.16]	0.08	[0.01, 0.15]	*p = 0.034*
CISPLATIN	1.05	[0.96, 1.15]	0.05	[−0.05, 0.14]	*p* = 0.326
OXALIPLATIN	0.87	[0.79, 0.95]	−0.14	[−0.24, −0.05]	*p =* 0.003

Reports submitted by HCPs included physicians, pharmacists, nurses, health professionals, and other HCPs, as defined by occupation codes in the DEMO table.

**Table 5:** Structure of the 2 × 2 contingency table used for disproportionality analysis in the FAERS database.

	Alopecia (+)	Alopecia (−)
Reports with suspected drug	a	b
All other reports	c	d

Reporting odds ratio (ROR) = (a × d)/(b × c).

## Data Availability

The data analyzed in this study are publicly available from the FDA Adverse Event Reporting System.

## Supplementary Materials

The following supporting information can be downloaded at: https://www.mdpi.com/article/10.3390/ph19030445/s1, Table S1: Preferred Terms included in the MedDRA High Level Term “Alopecias” used for the disproportionality analysis; Table S2: Reporter type-specific counts of alopecia cases for the top 25. Supplementary Figure S1: Temporal Changes in Reporter Composition for Docetaxel-Associated Alopecia in the United States (2005–2024); Supplementary Figure S2: Quarterly Report Counts of Docetaxel-Associated Alopecia by Reporter Group in the United States (2005–2024).

## Abbreviations

The following abbreviations are used in this manuscript:

FAERS	FDA Adverse Event Reporting System
SRS	Spontaneous reporting system
AE	Adverse event
MedDRA	Medical Dictionary for Regulatory Activities
HLT	High-Level Term
PT	Preferred Term
LLT	Lowest-Level Term
ATC	Anatomical Therapeutic Chemical Classification System
HCPs	Healthcare professionals
ROR	Reporting odds ratio
lnROR	Natural logarithm of the reporting odds ratio
CI	Confidence interval
IQR	Interquartile range
CTIA	Cancer therapy-induced alopecia
CIA	Chemotherapy-induced alopecia
EIA	Endocrine therapy-induced alopecia
pCIA	Permanent chemotherapy-induced alopecia
QoL	Quality of life
HRQoL	Health-related quality of life
