# Supplementary material for: Impact of Reporter Type on Signal Detection of Cancer Therapy-Induced Alopecia: A Hypothesis-Generating Study Using the FDA Adverse Event Reporting System
Source: Pharmaceuticals (Basel). 2026 Mar 10;19(3):445. doi: 10.3390/ph19030445 (PMC13029735; doi:10.3390/ph19030445)
Supplement: Supplementary file 1 [file pharmaceuticals-19-00445-s001.zip › Supplementary Figures S1.pdf]

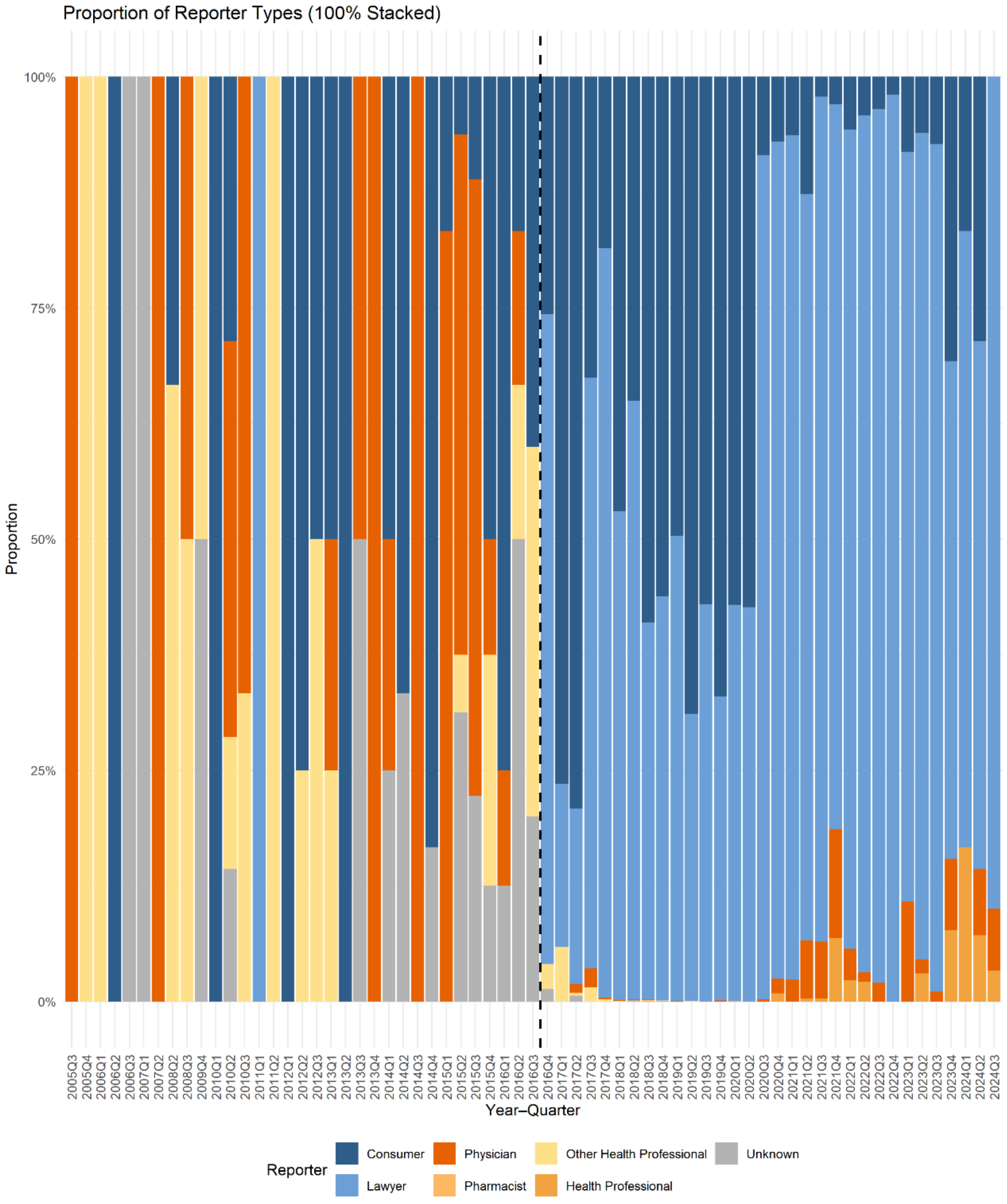

Supplementary Figure S1. Temporal Changes in Reporter Composition for Docetaxel-Associated Alopecia in the United States (2005–2024).

Supplementary Figure S1. Temporal Changes in Reporter Composition for Docetaxel-Associated Alopecia in the United States (2005–2024).

Proportion of reporter types per quarter (100% stacked bar plot) for U.S.-reported FAERS cases in which docetaxel was recorded as a primary or secondary suspect drug and alopecia-related PTs were reported. The dashed vertical line (2016 Q4) indicates the onset of U.S. mass litigation related to docetaxel. Following this period, reports from non-healthcare professionals (consumers and lawyers) increased markedly in proportional terms, whereas reports from healthcare professionals remained comparatively stable.

Reporter group definitions (FAERS occupation codes):
Healthcare professionals (HCPs) = MD (physician), PH (pharmacist), OT (other health professional), HP (health professional not otherwise specified).
Non-healthcare professionals (Non-HCPs) = CN (consumer), LW (lawyer), and unknown reporters (n = 31).
† RN (registered nurse) was not observed in this subset (count = 0) and is therefore not shown; HCP proportions are computed over MD/PH/OT/HP only.
